# An Improved Ensemble of Random Vector Functional Link Networks Based on Particle Swarm Optimization with Double Optimization Strategy

**DOI:** 10.1371/journal.pone.0165803

**Published:** 2016-11-11

**Authors:** Qing-Hua Ling, Yu-Qing Song, Fei Han, Dan Yang, De-Shuang Huang

**Affiliations:** 1 School of Computer Science and Communication Engineering, Jiangsu University, Zhenjiang, China; 2 School of Computer Science and Engineering, Jiangsu University of Science and Technology, Zhenjiang, China; 3 School of Electronics and Information Engineering, Tongji University, Shanghai, China; Beihang University, CHINA

## Abstract

For ensemble learning, how to select and combine the candidate classifiers are two key issues which influence the performance of the ensemble system dramatically. Random vector functional link networks (RVFL) without direct input-to-output links is one of suitable base-classifiers for ensemble systems because of its fast learning speed, simple structure and good generalization performance. In this paper, to obtain a more compact ensemble system with improved convergence performance, an improved ensemble of RVFL based on attractive and repulsive particle swarm optimization (ARPSO) with double optimization strategy is proposed. In the proposed method, ARPSO is applied to select and combine the candidate RVFL. As for using ARPSO to select the optimal base RVFL, ARPSO considers both the convergence accuracy on the validation data and the diversity of the candidate ensemble system to build the RVFL ensembles. In the process of combining RVFL, the ensemble weights corresponding to the base RVFL are initialized by the minimum norm least-square method and then further optimized by ARPSO. Finally, a few redundant RVFL is pruned, and thus the more compact ensemble of RVFL is obtained. Moreover, in this paper, theoretical analysis and justification on how to prune the base classifiers on classification problem is presented, and a simple and practically feasible strategy for pruning redundant base classifiers on both classification and regression problems is proposed. Since the double optimization is performed on the basis of the single optimization, the ensemble of RVFL built by the proposed method outperforms that built by some single optimization methods. Experiment results on function approximation and classification problems verify that the proposed method could improve its convergence accuracy as well as reduce the complexity of the ensemble system.

## Introduction

Neural network ensemble (NNE) is a learning mechanism which has a collection of a finite number of neural networks trained for the same task [1]. Much work has shown that ensemble-based machine learning approaches to classification could outperform canonical single-predictor classifiers [2, 3]. By combining a set of so-called base classifiers, the deficiencies of each classifier may be compensated by the efficiency of the others [4]. In the past decades, neural network ensemble has gained widespread interest among researchers in machine learning community.

Traditional neural network ensemble usually selects backpropagation (BP) and radial basis function (RBF) network models [5] as the base classifiers in many cases. Although these ensembles of neural networks could obtain higher convergence accuracy than many single classifiers, it is quite difficult to determine a suitable network structure and some parameters in each base classifier. Moreover, the base classifiers require thousands of iterations to learn the input to output relation in the given data, so the learning process of the base classifiers is time consuming.

To overcome the defects of BP based learning algorithms, random vector functional link networks (RVFL) was proposed [6] where actual values of the weights from the input layer to hidden layer can be randomly generated in a suitable domain and kept fixed in the learning stage [7]. Randomization has been getting increasing attention in the area of machine learning, mostly thanks to the resulting simplicity and speed in the empirical training process [8, 9]. The RVFL is a universal approximator for a continuous function on a bounded finite dimensional set with a closed-form solution [10], and it has been employed to solve problems in diverse domains [8]. The independently developed method, single hidden layered feedforward neural networks with random weights (RWSLFN) in [11] without direct links between the inputs and outputs, belongs to the family of RVFL. The experiment results in [8] verified that the direct links between the inputs and outputs led to slightly better performance than RWSLFN in all cases. However, RWSLFN has the potential of achieving better generalization performance because of its simple network structure, and it also requires less computational cost than those with the direct links. Moreover, a slight improvement on convergence accuracy of base classifiers would not surely improve the convergence accuracy of ensemble system. Therefore, this study focuses on RWSLFN ensemble.

As an effective learning algorithm for RWSLFN, extreme learning machine (ELM) [12] has been widely used in various applications [13], which randomly chooses the input weights and hidden biases and analytically determines the output weights of single hidden layered feedforward neural networks (SLFN). Different from traditional iterative learning algorithm for RWSLFN, ELM not only has faster learning speed but also achieves better generalization performance [14, 15]. Moreover, non-differentiable activation functions and straightforward solution are features and advantages of ELM. However, for randomly selecting the input weights and hidden biases, the uncertainty performance and over-fitting of ELM still remain to be solved [16, 17].

According to the above discussion, it is necessary and possible to select extreme learning machine as the base classifiers in the neural network ensemble. To overcome the deficiencies of single ELM and build an effective neural network ensemble, some ensemble of ELM were proposed. In [18], Liu et al. proposed an ensemble of ELM (E-ELM) which embedded cross-validation into the training phase for alleviating the overtraining problem and increasing the predictive stability. In [19], an ELM ensemble was proposed to investigate the interactions of different inducing factors affecting the evolution of landslide, which provided a good representation of the measured slide displacement behavior for the real data. In [20], an ensemble of online sequential extreme learning machine (EOS-ELM) was proposed to enhance the stability of online sequential ELM. Tian et al. [21, 22] introduced Bagging and AdaBoost methods to combine ELM to establish regression prediction model. In [23], an ensemble of ELM, called LSTD-eELM, was proposed for value prediction in continuous-state problems. RMSE-ELM, proposed in [24], recursively employed selective ensemble to pick out several optimal ELM from bottom to top for the final ensemble. The experiments verified that the robustness performance of RMSE-ELM was better than original ELM and some representative methods for blended data.

Because of their better optimization performance, some evolutionary computation techniques such as genetic algorithm (GA) [25] and particle swarm optimization (PSO) [26] are used to build neural network ensemble. Zhou et al. [27] introduced GA based selective ensembles (GASEN), which trained several individual neural networks and then employed GA to select an optimum subset of individual neural networks to constitute an ensemble. In [4], a multi-objective genetic programming approach to evolving accurate and diverse ensembles of genetic program classifiers with good performance on both the minority and majority of classes was proposed. In [28], an evolutionary approach named as EE-ELM was proposed for constituting ELM ensembles. EE-ELM employed the model diversity as fitness function to direct the selection of base learners, and produced an optimal solution with ensemble size control [28]. The experiment results demonstrated that the EE-ELM method outperformed some ensemble techniques including simple average, bagging and AdaBoost, in terms of both effectiveness and efficiency [28]. Compared with GA, PSO has its advantages such as easy to implement, few parameters and fast convergence rate [29–32]. These advantages make it suitable to employ PSO to establish ensembles. In [33], a PSO based selective neural network ensemble (PSOSEN) algorithm was proposed, which was used for the Nasdaq-100 index of Nasdaq Stock MarketSM and the S&P CNX NIFTY stock index analysis. In [34], PSO was used to optimize the weights associated to each base classifier, which showed the stable and improved performance on the selected datasets.

However, traditional PSO has the drawbacks of premature convergence and easily falling into local minima [29, 35]. To avoid premature convergence effectively in the search process, an improved PSO called attractive and repulsive particle swarm optimization (ARPSO) [36] was proposed, which could obtain better search performance than traditional PSO by adaptively controlling the diversity of the swarm. In [37], we proposed an ensemble of ELM based on ARPSO (E-ARPSOELM) which used ARPSO to select the base ELM by considering the convergence accuracy of the ensemble system. In E-ARPSOELM [37], the ensemble weights were simply calculated according to the validation accuracies of all selected ELM. Based on the E-ARPSOELM, we proposed a diversity guided ensemble of ELM based on ARPSO (DGEELMBARPSO) [38] which used ARPSO to select the base ELM from the initial ELM pool by considering both the classification accuracy and diversity of the ensemble system represented by each particle. The DGEELMBARPSO method used the simple majority voting and weighting voting methods as the decision rules. Experiment results verified that these ARPSO based ELM ensembles obtained better convergence performance than some PSO based and classical ELM ensembles.

In this paper, we further propose an improved ARPSO-based ELM ensemble by using ARPSO to select and combine the base ELM. Different form the DGEELMBARPSO, the new method uses ARPSO to perform double optimization in two phases. In the first phase, a modified ARPSO is employed to select the base ELM from the initial ELM pool by considering the convergence accuracy and diversity of the candidate ensemble system, which is the same to the DGEELMBARPSO method. In the second phase, we use ARPSO to optimize the ensemble weights related to the selected ELM in this study, while the ensemble weights in [37, 38] were determined directly without any optimization. In this study, the initial ensemble weights related to the selected ELM are obtained by the minimum norm least-square (LS) method firstly. Then, with the initial ensemble weights, the traditional ARPSO is used to optimize the ensemble weights. Finally, theoretical analysis and justification on how to prune redundant base ELM in the ensemble system is presented for classification problems, and a practically feasible pruning strategy is proposed in the proposed approach for both classification and regression problems. The proposed approach could not only further improve the convergence accuracy of the ensemble system but also reduce the redundancy of the ensemble system. Experiment results on function approximation, four benchmark classification problems from UCI Repository database and two microarray data have verified the effectiveness of the proposed method.

The remainder of this paper is organized as follows. Section 2 introduces the related methods including ELM and ARPSO algorithms. The improved ensemble of ELM is proposed in Section 3. In Section 4, experiment results and discussion on seven data are given to verify the efficiency and effectiveness of the proposed approach. Finally, the concluding remarks are offered in Section 5. There are a lot of the abbreviations in this paper. For ease of understanding, all the abbreviations and their paraphrases are listed in Table 1.

**Table 1:** Abbreviation comparison table.

Abbreviation	Paraphrase	Abbreviation	Paraphrase
NNE	neural network ensemble	BP	backpropagation
RBF	radial basis function	RVFL	random vector functional link networks
SLFN	single hidden layered feedforward neural networks	RWSLFN	SLFN with random weights
ELM	extreme learning machine	E-ELM	an ensemble of ELM proposed in [18]
EOS-ELM	an ensemble of online sequential ELM	LSTD-eELM	an ensemble of ELM proposed in [23]
RMSE-ELM	an ensemble of ELM proposed in [24]	GA	genetic algorithm
PSO	particle swarm optimization	GASEN	GA based selective ensembles
EE-ELM	an ensemble of ELM proposed in [28]	PSOSEN	PSO based selective NNE
ARPSO	attractive and repulsive PSO	E-ARPSOELM	an ensemble of ELM proposed in [37]
DGEELMBARPSO	an ensemble of ELM proposed in [38]	MP	Moore-Penrose
LS	least square	APSO	adaptive PSO
DO-EELM	an ensemble of ELM based on double optimization	RMSE	root mean squared error
E-PSOELM	an ensemble of ELM based on PSO	SO-EELM	an ensemble of ELM proposed in [38]

## Preliminaries

### Extreme learning machine

In [12], a learning algorithm for SLFN called extreme learning machine (ELM) was proposed to solve the problem caused by gradient-based learning algorithms. ELM randomly chose the input weights and hidden biases, and analytically determined the output weights of SLFN. ELM has much better generalization performance with much faster learning speed than gradient-based algorithms [39, 40].

For *N* arbitrary distinct samples, (*x*_*i*_, *t*_*i*_), where *x*_*i*_ = [*x*_*i*1_, *x*_*i*2_, … *x*_*in*_]^*T*^ ∈ *R*^*n*^, *t*_*i*_ = [*t*_*i*1_, *t*_*i*2_, …, *t*_*im*_]^*T*^ ∈ *R*^*m*^, a SLFN with *N*_*H*_ hidden neurons can approximate these *N* samples with zero error. This means that

Hwo=T (1)

where $H({wh}_{1},\ldots ,{wh}_{N_{H}},b_{1},\ldots ,b_{N_{H}},x_{1},\ldots ,x_{N})$

$$={\left[\begin{array}{ccc} g(wh_{1}\cdot x_{1}+b_{1}) & \cdots & g(wh_{N_{H}}\cdot x_{1}+b_{N_{H}})\\ \vdots & \ldots & \vdots \\ g(wh_{1}\cdot x_{N}+b_{1} & \ldots & g(wh_{N_{H}}\cdot x_{N}+b_{N_{H}}) \end{array}\right]}_{N\times N_{H}},\;wo={\left[\begin{array}{c} wo_{1}^{T}\\ \vdots \\ {wo}_{N_{H}}^{T} \end{array}\right]}_{N_{H}\times m},\;T={\left[\begin{array}{c} t_{1}^{T}\\ \vdots \\ t_{N}^{T} \end{array}\right]}_{N\times m}.$$

*wh*_*i*_ = [*wh*_*i*1_, *wh*_*i*2_, …, *wh*_*in*_]^*T*^ is the weight vector connecting the *i*-th hidden neuron to the input neurons, *wo*_*i*_ = [*wo*_*i*1_, *wo*_*i*2_, …, *wo*_*im*_]^*T*^ is the weight vector connecting the *i*-th hidden neuron to the output neurons, *b*_*i*_ is the bias of the *i*-th hidden neuron, and g(⋅) is the activation function of hidden neurons.

Thus, to determine the output weights is to find the least square (LS) solution to the given linear system. The minimum norm LS solution to the linear system (1) is

$$w\;o=H{\;}^{+}T$$ (2)

where *H*^*+*^ is the Moore-Penrose (MP) generalized inverse of matrix *H*. The minimum norm LS solution is unique and has the smallest norm among all the LS solutions. ELM using such MP inverse method tends to obtain good generalization performance [16]. Since the solution is obtained by an analytical method and all the parameters of SLFN need not be adjusted, ELM converges much faster than gradient-based algorithms.

### Particle swarm optimization

PSO is an evolutionary computation technique in search of the best solution by simulating the movement of birds in a flock [26]. The population of the birds is called swarm, and the members of the population are particles. Each particle represents a possible solution to the optimization problem. During each iteration, each particle flies independently in its own direction which is guided by its own previous best position as well as the global best position of all the particles. Assume that the dimension of the search space is *R*, and the swarm is *S = (X*_*1*_, *X*_*2*,_ *X*_*3*_, *…*, *X*_*Np*_*)*; each particle represents a position in *R* dimension space; the position of the *i*-th particle in the search space is denoted as *X*_*i*_ *= (x*_*i1*_, *x*_*i2*,_ *…*, *x*_*iR*_*)*, *i* = *1*, *2*, *…*, *N*_*p*_, where *N*_*p*_ is the size of the swarm. The previous best position of the *i*-th particle is called *pbest* which is expressed as *P*_*i*_ *= (p*_*i1*_, *p*_*i2*,_ *…*, *p*_*iR*_*)*. The best position of the all particles are called *gbest* which is expressed as *P*_*g*_ *= (p*_*g1*_, *p*_*g2*,_ *…*, *p*_*gR*_*)*. The velocity of the *i*-th particle is expressed as *V*_*i*_ *= (v*_*i1*_, *v*_*i2*_, *…*, *v*_*iR*_*)*. According to [26], the basic PSO was described as:

$$V_{i}(t+1)=V_{i}(t)+c_{1}\times rand()\times (P_{i}(t)-X_{i}(t))+c_{2}\;\times rand()\times (P_{g}(t)-X_{i}(t))$$ (3)

$$X_{i}(t+1)=X_{i}(t)+V_{i}(t+1)$$ (4)

where *c*_*1*_, *c*_*2*_ are the acceleration constants with positive values; *rand()* is a random number ranged from 0 to 1.

To obtain better performance, an improved PSO called adaptive PSO (APSO) was proposed [41], and the corresponding velocity update of particles was denoted as follows:

$$V_{i}(t+1)=W(t)\times V_{i}(t)+c_{1}\times rand()\times (P_{i}(t)-X_{i}(t))+c_{2}\;\times rand()\times (P_{g}(t)-X_{i}(t))$$ (5)

where *W(t)* is the inertia weight to keep a balance between global search and local search. The inertia weight can be computed by the following equation:

$$W(t)=W_{\text{max}}-t\times (W_{\text{max}}-W_{\text{min}})/N_{PSO}$$ (6)

In Eq (6), *W*_*max*_, *W*_*min*_ and *N*_*pso*_ are the initial inertial weight, the final inertial weight and the maximum optimization iterations, respectively.

Although PSO has shown good performance in solving many optimization problems, it suffers from the problem of premature convergence like most of the stochastic search techniques, particularly in multimodal optimization problems [42]. To overcome premature convergence of PSO, in [36], attractive and repulsive particle swarm optimization (ARPSO), a diversity guided method, was proposed which was described as:

$$V_{i}(t+1)=V_{i}(t)+dir\times [c_{1}\times rand()\times (P_{i}(t)-X_{i}(t))+c_{2}\;\times rand()\times (P_{g}(t)-X_{i}(t))]$$ (7)

where $dir=\{\begin{array}{ll} -1 & diversity<d_{low}\\ 1 & diversity>d_{high} \end{array}$.

In [36], a function was proposed to calculate the diversity of the swarm as follows:

diversity()=(1Np×|L|)×∑i=1Np∑j=1R(pij−pj¯)2 (8)

where *|L|* is the length of the maximum radius of the search space; *p*_*ij*_ is the *j*-th component of the *i*-th particle and $\bar{p_{j}}$ is the average of the *j*-th component over all particles.

In the attraction phase (*dir = 1*), the swarm is attracting, and consequently the diversity decreases. When the diversity drops below the lower bound, *d*_*low*_, the swarm switches to the repulsion phase (*dir = -1*) when the swarm is repelling. When the diversity reaches the upper bound, *d*_*high*_, the swarm switches back to the attraction phase. ARPSO alternates between phases of exploiting and exploring—attraction and repulsion—low diversity and high diversity and thus improve its search ability [36].

## The Improved Ensemble of RVFL Based on Double Optimization

### The proposed method (DO-EELM)

**Fig 1:**
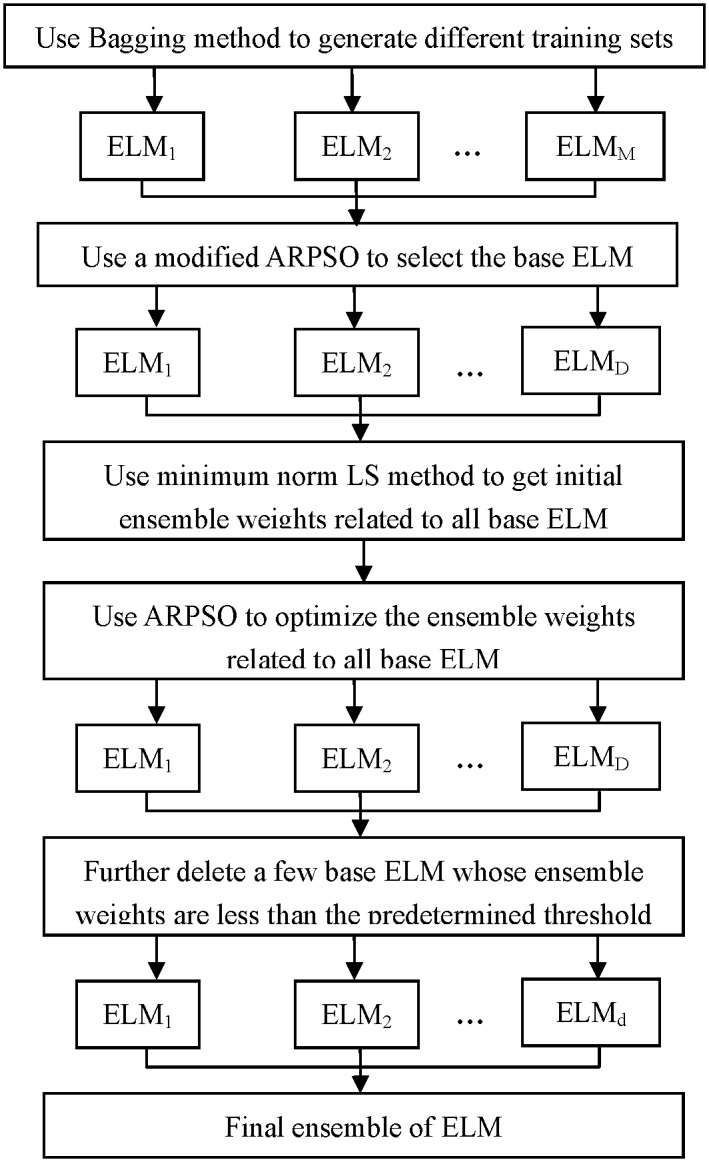
The frame of the DO-EELM method.

The critical steps to build an ensemble system include how to select the optimal base classifiers and how to determine the ensemble weights for the selected base classifiers. In this study, double optimization is performed by ARPSO to select the base ELM and optimize the ensemble weights corresponding to each selected ELM. As for selecting the base classifiers, we use APRSO to search the optimal ELM sets by considering both the classification performance and diversity of the ensemble system, which makes the ensemble system gain high convergence ability with comparatively high diversity. To obtain the optimal ensemble weights, the initial weights of the selected base ELM are determined by the minimum norm LS method, and then are further optimized by ARPSO. Moreover, to reduce the complexity of the ensemble system without influencing the convergence accuracy of the ensemble system, a few base ELM with much lower ensemble weights than others is pruned from the ensemble system. Since the proposed ensemble of RVFL is built on ELM with double optimization based on ARPSO, it is referred to as the DO-EELM. The rough framework of the DO-EELM is shown in Fig 1.

The DO-EELM builds the effective ensemble system with two phases. In the first phase, a modified ARPSO is used to select the optimal base ELM. The detailed steps are described as follows:

Step A1: Form an initial ELM pool. The dataset is divided into the training and testing datasets. On the training datasets, the Bagging method [43] is used to randomly assign different sub-training datasets with the same size. With a sub-training dataset, a corresponding ELM is randomly generated to train a SLFN. All ELM forms the initial ELM pool for further selection, and they have the same number of hidden nodes. Moreover, the original training datasets are further divided into the training and validation datasets.

Step A2: Initialize the swarm. Randomly initialize the position, *X*_*i*_ *= (x*_*i1*_, *x*_*i2*,_ *…*, *x*_*iM*_*)*, *i* = *1*, *2*, *…*, *N*_*p*_, and the velocity, *V*_*i*_ *= (v*_*i1*_, *v*_*i2*_, *…*, *v*_*iM*_*)*, of each particle, where *M* is the number of the initial ELM pool and *N*_*p*_ is the warm size. The value of the component of the *i*-th particle, *x*_*ij*_, is rounded as 1 or 0 which indicates the *j*-th ELM be selected or not to build the ensemble system.

Step A3: Select the optimal base ELM subsets by the modified ARPSO.

Substep A3.1: Set *X*_*i*_ as the current *pbest* for the *i*-th particle, compute fitness values of all particles, and find the global best position *gbest*. For regression problem, the fitness function is the negative root mean squared error (RMSE) on the validation dataset. As for classification problem, the fitness function is defined as the classification accuracy on the validation datasets obtained by the ensemble system represented by the particle. The fitness function of the *i*-th particle for classification problems is defined as follows:

$$f(X_{i})=\sum_{k=1}^{N_{v}}\left(T_{k}•Y_{k}^{i}\right)/N_{v}$$ (9)

where *N*_*v*_ is the number of samples in the validation datasets, $Y_{k}^{i}$ and *T*_*k*_ are the actual output of the *i*-th ensemble system and the desired output for the *k*-th sample. When $Y_{k}^{i}$ is equal to *T*_*k*_, their inner product, $T_{k}•Y_{k}^{i}$, is equal to 1.

Substep A3.2: Update *V*_*i*_ and *X*_*i*_ according to Eqs (7) and (4), respecively. At the same time, *x*_*ij*_ needs rounding operation and new population is generated. If *x*_*ij*_ is greater than 1, it will be set as 1.

Substep A3.3: Calculate new fitness values of all particles, and update the *pbest* and *gbest* for all particles. To obtain the ensemble system with improved diversity, the *pbest* and *gbest* are updated according to Eqs (10) and (11), respectively.

$$P_{i}=\{\begin{array}{cc} X_{i} & \left((f(X_{i})-f(P_{i}))>\alpha )\;or\;(|f(X_{i})\;-f(P_{i})|\;<\alpha \;and\;div(X_{i})>div(P_{i})\right)\\ P_{i} & \;else \end{array}$$ (10)

$$P_{g}=\{\begin{array}{cc} X_{i} & \left((f(X_{i})-f(P_{g}))>\alpha )\;or\;(|f(X_{i})\;-f(P_{g})|<\alpha \;and\;div(X_{i})>div(P_{g})\right)\\ P_{g} & \;else \end{array}$$ (11)

*f(X*_*i*_*)*, *f(P*_*i*_*)* and *f(P*_*g*_*)* are the fineness values of the *i*-th particle, the *pbest* of the *i*-th particle and the *gbest* of the swarm, respectively; *div(X*_*i*_*)*, *div(P*_*i*_*)* and *div(P*_*g*_*)* are the diversity of the ensemble system represented by the *i*-th particle, the *pbest* of the *i*-th particle and the *gbest* of the swarm, respectively.

The diversity is an important factor of ensemble algorithms and there is no agreed definition for diversity [44]. Assume that the each base ELM was a point in the space. An ELM could be represented by its input weights, hidden biases, output weights and corresponding output for a training sample. It is evident that the greater the distance between two ELM is, the greater the difference between the two ELM is. The diversity of the ensemble system represented by the *i*-th particle, *div(X*_*i*_*)*, is defined as follows:

div(Xi)=2∑k=1Ni−1∑l=k+1Ni(‖WHk−WHl‖22+‖Bk−Bl‖22+‖WOk−WOl‖22+‖Yk−Yl‖22)Ni×(Ni−1) (12)

where *WH*_*k*_ and *WH*_*l*_ are the input weights matrices of the *k*-th and *l*-th selected ELM, respectively, in the *i*-th particle, *B*_*k*_ and *B*_*l*_ are the hidden biases vectors of the *k*-th and *l*-th selected ELM, respectively, in the *i*-th particle, *WO*_*k*_ and *WO*_*l*_ are output weights matrices of the *k*-th and *l*-th selected ELM, respectively, in the *i*-th particle, and *Y*_*k*_ *= (Y*_*k1*_, *Y*_*k2*_, *…*, *Y*_*kNtrain*_*)* and *Y*_*l*_ *= (Y*_*l1*_, *Y*_*l2*_, *…*, *Y*_*lNtrain*_*)* are the actual output vectors of the *k*-th and *l*-th selected ELM, respectively, in the *i*-th particle on all training data. *N*_*i*_ and *Ntrain* are the number of the base ELM in the ensemble system represented by the *i*-th particle and the size of the training datasets.

Substep A3.4: The above optimization process from Substep A3.2 to Substep A3.3 is repeated until the goal is met or the maximum optimization epochs are completed.

In the second phase, ARPSO is used to optimize the ensemble weights related to the selected base ELM obtained in the first phase. The detailed steps are described as follows.

Step B1: Initialize the ensemble weights for all selected base ELM by the minimum norm LS method. Assume that

$$\tilde{Y}\tilde{W}=T_{Ntrain}$$ (13)

where $\tilde{Y}={\left[\begin{array}{ccc} {\tilde{Y}}_{1,1} & \cdots & {\tilde{Y}}_{1,D}\\ \vdots & \ldots & \vdots \\ {\tilde{Y}}_{Ntrain,1} & \ldots & {\tilde{Y}}_{Ntrain,D} \end{array}\right]}_{Ntrain\times m\times D},\;\tilde{W}={\left[\begin{array}{c} {\tilde{W}}_{1}\\ \vdots \\ {\tilde{W}}_{D} \end{array}\right]}_{D\times 1}^{T},\;T_{Ntrain}={\left[\begin{array}{c} t_{1}^{T}\\ \vdots \\ t_{Ntrain}^{T} \end{array}\right]}_{Ntrain\times m}$.

${\tilde{Y}}_{i,j}$ is the output of the *j*-th ELM for the *i*-th training sample, ${\tilde{W}}_{i}$ is the ensemble weight for the *i*-th base ELM, and *D* is the number of the selected ELM in the first phase. By the minimum norm LS method, the initial ensemble weights are calculated as follows:

$$\tilde{W}={\left(\tilde{Y}\right)}^{+}T_{Ntrain}$$ (14)

Step B2: Use ARPSO to optimize the ensemble weights with the initial values obtained by Eq (14). The optimization process in this phase is similar to that in the first phase, but some details should be clarified. First, as for initializing the swarm, one particle is initialized as the values obtained by Eq (14), and the other particles are randomly initialized within the values in the interval of (0, 1). Second, the fitness function is also the corresponding classification accuracy and negative RMSE on the validation dataset for classification and regression problems, respectively. Finally, the *pbest* and *gbest* are updated as those updated in the traditional PSO, which is different from that in the first phase.

Step B3: Delete a few redundant base ELM without influencing the convergence accuracy of the ensemble system.

Step B4: The optimal ensemble of ELM is obtained, and then applied to the test dataset.

From the DO-EELM approach, the following conclusion can be concluded.

First, in the process of selecting the base ELM, ARPSO considers not only the classification accuracy on validation dataset but also the diversity of the corresponding ensemble system, so the DO-EELM could obtain the ensemble of ELM with improved classification ability and diversity.

Second, after searching the optimal base ELM subset which gains the best generalization performance with comparatively high diversity in the process of the first optimization, the DO-EELM further search the optimal ensemble weights to build the best ensemble of ELM in the second optimization. Since the double optimization is performed based on the single optimization, the DO-EELM could achieve the better convergence performance than those ensembles of ELM with single optimization strategy. Moreover, the DO-EELM could build more compact ensemble of ELM without decreasing the convergence performance because of pruning the redundant base ELM. According to [45], the smaller neural networks could obtain better generalization performance, so pruning the redundant base ELM from the ensemble system in the DO-EELM could further improve the generalization performance of the ensemble system.

Third, the DO-EELM selects the base ELM by ARPSO, so it could control the size of the ensemble system adaptively.

Finally, since the output weights for each ELM are analytically determined, the computational complexity of the DO-EELM is the same as that of ARPSO.

The computational complexity of the DO-EELM method can be calculated as follows:

$${CC}_{DO-EELM}=O(M)+O({Iter}_{arpso1}\times N_{arpso1}\times M)+O({Iter}_{arpso2}\times N_{arpso2}\times D)+O(D)$$ (15)

where *Iter*_*arpso1*_ and *N*_*arpso1*_ are the maximum iterations and swarm size of the ARPSO in the first phase, respectively; *Iter*_*arpso2*_ and *N*_*arpso2*_ are the maximum iterations and swarm size of the ARPSO in the second phase, respectively; *M* and *D* are the size of the initial ELM pool and the number of the selected ELM in the first phase. The four items on the right side of Eq (15) are the computational complexity of establishing initial ELM pool, selecting the base ELM by the modified ARPSO, optimizing the ensemble weights by the traditional ARPSO, and pruning the redundant base ELM from the ensemble system, respectively. Since *Iter*_*arpso1*_ and *Iter*_*arpso2*_ (*N*_*arpso1*_ and *N*_*arpso2*_) are the same order of magnitude and *M* is greater than *D*, the computational complexity of the DO-EELM method is approximated as *O*(*Iter*_*arpso*1_ × *N*_*arpso*1_ × *M*) which is the same as that of ARPSO.

The space complexity of the DO-EELM method can be calculated as follows:

$${SC}_{DO-EELM}=O(N_{sam})+O(M\times ((N_{in}+1)\times N_{h}+N_{h}\times N_{o})+O(N_{arpso1}\times 3M)+O(N_{arpso2}\times 3D)$$ (16)

where *N*_*sam*_ is the number of all samples in dataset; *N*_*in*_, *N*_*h*_, *N*_*o*_ are the number of input nodes, hidden nodes and output nodes, respectively, of the SLFN in each base ELM. The four items on the right side of Eq (16) are the space complexity of all samples, all ELM in the initial gene pool, the modified ARPSO in the first phase and the traditional ARPSO in the second phase, respectively. Similarly, Since *N*_*arpso1*_ and *N*_*arpso2*_ are the same order of magnitude and *M* is greater than *D*, the space complexity of the DO-EELM method is approximated as *O*(*N*_*sam*_) + *O*(*M* × ((*N*_*in*_ + 1) × *N*_*h*_ + *N*_*h*_ × *N*_*o*_ + *N*_*arpso*1_).

### Theoretical analysis and discussion of pruning the base ELM

Assume that *D* base ELM is selected by ARPSO, and the ensemble weight of the *i*-th base ELM is ${\tilde{W}}_{i},\;(i=1,\;2,\;\cdots \cdots ,\;D)$; The output of each base ELM is an *m*-dimensional vector where only one component is equal to one indicating the sample class, and the other components are zero. For the *l*-th training sample, the output of the *k*-th base ELM is ${\tilde{Y}}_{l,k}$, (*l* = 1, 2, …, *Ntrain*, *k* = 1, 2, …, *D*.), and the output of the ensemble system is ${\tilde{Y}}_{l}=\sum_{k=1}^{D}{\tilde{W}}_{k}\times {\tilde{Y}}_{l,k}$. Obviously, the *j*-th component of ${\tilde{Y}}_{l}$, can be represented as $\sum_{r=1}^{D}{\tilde{W}}_{j_{r}}$, and the *j*_*r*_ is defined as follows:

$$j_{r}=\{\begin{array}{cc} r & The\;r-th\;base\;ELM\;identifies\;the\;l-th\;sample\;as\;the\;j-th\;class.\\ 0 & else \end{array}$$ (17)

where ${\tilde{W}}_{0}$ is equal to zero. Moreover, the following equations are easily obtained.

$$\sum_{j=1}^{m}\sum_{r=1}^{D}{\tilde{W}}_{j_{r}}=\sum_{i=1}^{D}{\tilde{W}}_{i}$$ (18)

$$\left\{j_{r}\right\}\;\cap \;\left\{i_{r}\right\}=\emptyset ,\;i\neq j$$ (19)

For classification problem, if the value of the *k*-th component of ${\tilde{Y}}_{l}$ is the largest among those of all components, the ensemble system will identify the *l*-th training sample as the *k*-th class.

To prune the redundant base ELM is to delete those base ELM without influencing the classification result of the ensemble system. Assume that the *j*-th base ELM identifies the *l*-th sample as the *k*-th class. When the *j*-th base ELM is deleted, the value of the *k*-th component of the ensemble system is changed to $\sum_{r=1}^{D}{\tilde{W}}_{k_{r}}-{\tilde{W}}_{j}$. On one hand, if the ensemble system identifies the *l*-th sample as the *p*-th class where *p* is not same as *k*, to delete the *j*-th base ELM will not change the classification result of the ensemble system on the *l*-th sample because of $\sum_{r=1}^{D}{\tilde{W}}_{k_{r}}-{\tilde{W}}_{j}<\sum_{r=1}^{D}{\tilde{W}}_{k_{r}}<\sum_{r=1}^{D}{\tilde{W}}_{p_{r}}$. On the other hand, if the ensemble system identifies the *l*-th sample as the *k*-th class, to delete the *j*-th base ELM will not change the classification result of the ensemble system on the *l*-th sample when the value of the *k*-th component of the output vector of the ensemble system,$\sum_{r=1}^{D}{\tilde{W}}_{k_{r}}-{\tilde{W}}_{j}$, is still the largest among those of all output components of the ensemble system after deleting the *j*-th base ELM.

From the above analysis, when deleting the *j*-th base ELM does not change the classification results of the ensemble system on all the samples, the *j*-th base ELM is redundant and should be pruned. For classification problems, we can further draw the conclusion as follows.

**Theorem 1** Assume that *D* base ELM forms the ensemble system, and the ensemble weight of the *j*-th base ELM is ${\tilde{W}}_{j},\;(j=1,\;2,\;\cdots \cdots ,\;D)$; The sum of the ensemble weights of the *D* base ELM is equal to one. When the maximal values of the output components of the ensemble system on all samples are always greater than ${\tilde{W}}_{j}+0.5$, the *j*-th base ELM could be deleted without influencing the classification results of the ensemble system.

**Proof**: For simplicity, we consider the *l*-th sample firstly. The ensemble system identifies this sample as the *k*-th class, which means that the *k*-th component of the output vector of the ensemble system, $\sum_{r=1}^{D}{\tilde{W}}_{k_{r}}$, has the maximal value of all output components of the ensemble system.

According to the above analysis, if the *j*-th base ELM outputs different class on the *l*-th sample from the ensemble system, deleting the *j*-th base ELM certainly will not change the value of the *k*-th component of the output vector of the ensemble system.

Since the sum of the ensemble weights of the *D* base ELM is equal to one, the Eqs (18) and (19) has the form as follows:

$$\sum_{j=1}^{m}\sum_{r=1}^{D}{\tilde{W}}_{j_{r}}=1\;s.t.\;\left\{j_{r}\right\}\;\cap \;\left\{i_{r}\right\}=\emptyset ,\;i\neq j$$ (20)

If the *j*-th base ELM outputs the same class on the *l*-th sample from the ensemble system, deleting the *j*-th base ELM means that the value of the *k*-th component of the output vector of the ensemble system changes to $\sum_{r=1}^{D}{\tilde{W}}_{k_{r}}-{\tilde{W}}_{j}$. Since the maximal values of the output components of the ensemble system on all samples are always greater than ${\tilde{W}}_{j}+0.5$, the value of the $\sum_{r=1}^{D}{\tilde{W}}_{k_{r}}-{\tilde{W}}_{j}$ is greater than 0.5. According to Eq (20), the value of the $\sum_{r=1}^{D}{\tilde{W}}_{k_{r}}-{\tilde{W}}_{j}$ is still the largest value of all components of the output vector of the ensemble system, so the ensemble system still identifies the *l*-th sample as the *k*-th class after deleting the *j*-th base ELM.

Therefore, the *j*-th base ELM could be deleted without influencing the classification result on the *l*-th sample of the ensemble system.

The above proof also suits for other samples, and thus Theorem 1 is proved.

If the condition in Theorem 1, $\sum_{i=1}^{D}{\tilde{W}}_{i}=1$, is not satisfied, the condition will be realized by normalizing the ensemble weight of each base ELM as follows:

$${\hat{W}}_{j}=\frac{{\tilde{W}}_{j}}{\sum_{i=1}^{D}{\tilde{W}}_{i}},\;(j=1,2,\;\cdots \cdots ,\;D)$$ (21)

where ${\hat{W}}_{j}$ is the normalized ensemble weight of the *j*-th base ELM.

The above analysis and theorem provide a theoretical guide on how to prune the redundant base ELM for classification problems. However, the theoretical guide has two limitations which make the guide not suitable in most real cases. One is that the conditions of pruning the redundant base ELM are too rigorous, which can not be satisfied in most real cases. The other is that the above analysis and theorem only suit for classification problems but regression problems. For regression problem, the ultimate output of the ensemble system is the weighted sum of the output of all base ELM, so the above pruning method is not feasible.

Thus, we propose a more practically feasible strategy to prune the redundant base ELM in the DO-EELM which deletes the base ELM with much lower ensemble weights than others. When the ensemble weight of a base ELM is less than the predetermined threshold, *β*, the ELM will be pruned from the ensemble system. This strategy is feasible and flexible for both classification and regression problems. However, it is difficult to determine the value of the threshold. Generally, *β* is much less than the mean value of all ensemble weights, and the specific value should be determined by trial and error.

## Experiment Results and Discussion

In this section, to verify the effectiveness of the proposed approach, the DO-EELM is compared with E-ELM [18], EOS-ELM [20], E-PSOELM, E-ARPSOELM [37] and DGEELMBARPSO [38] on seven datasets. The E-PSOELM is similar to the E-ARPSOELM, while it uses adaptive PSO to select base ELM. Since the DGEELMBARPSO uses ARPSO to perform single optimization, we rename it SO-EELM in this section. We conduct experiments on one function approximation, four benchmark classification and two microarray data classification problems. Since support vector machine (SVM) is an effective learning algorithm with high performance, it is also compared with the proposed method in this section. The simulations for SVM on all data are carried out using compiled SVM package: LIBSVM which are available at http://www.csie.ntu.edu.tw/~cjlin/libsvm/. The kernel function used in SVM is radial basis function on all datasets. All the results shown in this paper are the mean values of 20 trials.

### Function approximation

In this section, all algorithms are used to approximate the ‘SinC’ function $y=\{\begin{array}{cc} \text{sin}(x)/x & x\neq 0\\ 1 & x=0 \end{array}$. A training set (*x*_*i*_, *y*_*i*_) and testing set (*x*_*j*_, *y*_*j*_) with 1000 data, respectively, are created where *x*_*i*_s and *x*_*j*_s are uniformly randomly distributed on the interval (-10,10). Moreover, large uniform noise distributed in [-0.2, 0.2] has been added to all the training samples while the testing data remains noise-free.

In the experiments, the number of the hidden nodes in all base ELM is 20. The activation functions of hidden nodes in all ELM are the sigmoid functions. For E-ELM and EOS-ELM, the number of the base ELM is fixed as twelve in the ensemble systems. As for ARPSO in the E-ARPSOELM, SO-EELM and DO-EELM on all datasets, the inertia weights *W*_*max*_ and *W*_*min*_ are set as 0.9 and 0.1, respectively; the parameters *d*_*low*_, *d*_*high*_, *c1* and *c2* are selected as 5e-6, 0.25, 2 and 2; the population size is 50; the size of the initial ELM pool is 40. The parameters values of basic PSO in E-PSOELM are same as those of ARPSO in ARPSO based ensemble of ELM. The parameter, *α*, is selected as 0.0005 in the SO-EELM and DO-EELM, and the threshold, *β*, is selected as 0.05 in the DO-EELM. These parameters are determined by trial and error and according to the guidance given in [29, 30, 41]. The corresponding results are shown in Table 2. The results of SVM are directly cited from the literature [46].

**Table 2:** The results of approximating the Sinc function by the seven algorithms.

Algorithms	Train RMSE	Test RMSE±Std.	Mean size of ELM ensemble
SVM	0.1149	0.0130±0.0012	/
E-ELM	0.1157	0.0166±6.7086e-04	12
E-OSELM	0.1163	0.0167±6.3027e-04	12
E-PSOELM	0.1164	0.0163±8.6240e-04	10.3
E-ARPSOELM	0.1153	0.0161±8.6240e-04	9.7
SO-EELM	0.1161	0.0133±6.8036e-04	9.6
DO-EELM	0.1156	0.0113±5.7065e-04	6.83

From Table 2, the DO-EELM method obtains the least test RMSE with the least number of ELM than the other ELM ensembles, which indicates that the proposed method could build more compact ensemble system with better generalization performance than the other ensemble of ELM. SVM obtains less test RMSE than other ELM ensembles but the DO-EELM. With the similar train RMSE, different test RMSE indicates different generalization performance of the ELM ensembles. For example, the train RMSE values of the E-ELM and DO-EELM are almost the same, but the test RMSE values of the E-ELM and DO-EELM vary greatly. This result demonstrates that the ensemble of ELM built by the DO-EELM has much better generalization performance than that built by the E-ELM.

**Fig 2:**
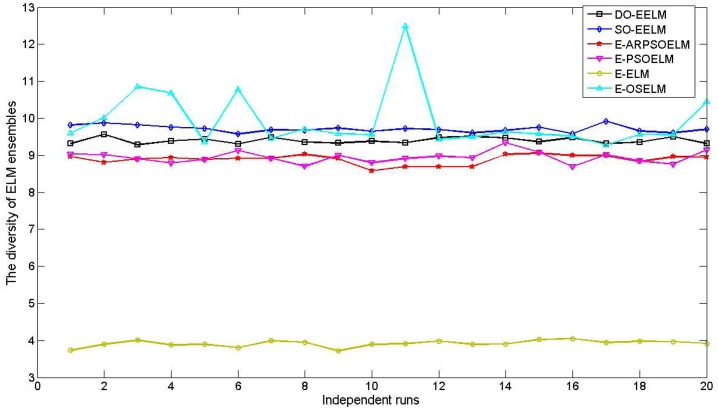
The diversity curves of different ensembles of ELM on approximating the SinC function with 20 independent runs.

Fig 2 shows the diversity values of different ensemble of ELM with 20 independent runs. The ensemble system built by the DO-EELM has higher diversity than those built by the E-ELM, E-PSOELM and E-ARPSOELM in all runs. The diversity of the ensemble system built by the DO-EELM is less than that of the E-OSELM and SO-EELM, which lies mainly in the fact that the ensemble system built by the DO-EELM has fewer ELM members than that of the E-OSELM and SO-EELM. Therefore, the proposed method could build the ensemble system with comparatively high diversity.

**Fig 3:**
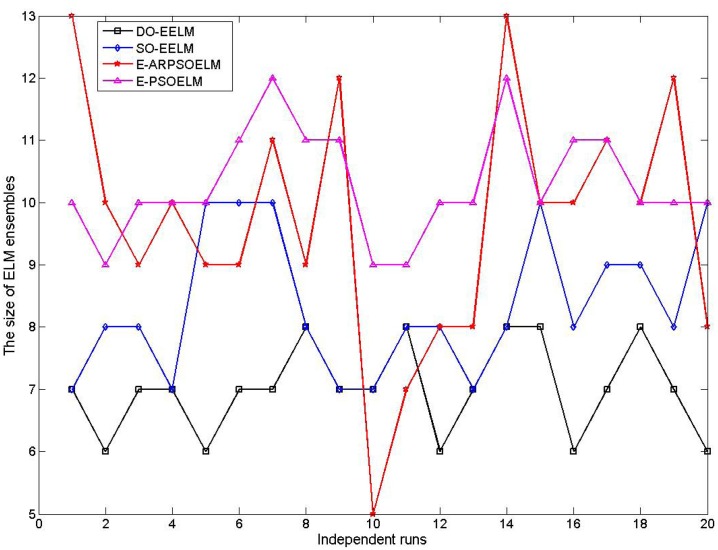
The number of the ELM in the four PSO based ensembles of ELM on approximating the SinC function with 20 independent runs.

The number of ELM in the E-ELM and E-OSELM is predetermined by trial and error, while the one in the E-PSOELM, E-ARPSOELM, SO-EELM and DO-EELM is determined adaptively in the selection process. Fig 3 shows the ELM number curves in the four PSO based ensemble of ELM with 20 independent runs. The DO-EELM selects least number of ELM of all PSO based ELM ensembles in most of runs, which indicates that the DO-EELM could establish more compact ensemble system than other ELM ensembles.

### Classification problems

In this subsection, the performance of the DO-EELM method is tested on the four benchmark classification problems from UCI Machine Repository Database (http://archive.ics.uci.edu/ml/) including Diabetes, Satellite Image, Wine and Image Segmentation data, and two microarray data which is hard to classify including Lung and Brain cancer data. The Lung data is available at http://www.genome.wi.mit.edu/MPR/lung and http://www.pnas.org, and the Brain cancer data is available at http://linus.nci.nih.gov/~brb/DataArchive_New.html. The specifications of the six data are presented in Table 3. For the Diabetes data, we use the “Pima Indians Diabetes Database” produced in the Applied Physics Laboratory, Johns Hopkins University, 1988. Moreover, the training sets and validation sets occupy 70% and 30% of the whole training sets, respectively.

**Table 3:** The specifications of the six datasets.

Dataset	Train set	Test set	Categories	Attributes
Diabetes	576	192	2	8
Satellite Image	4435	2000	6	36
Wine	120	58	3	13
Image Segmentation	1500	810	7	19
Brain cancer	41	19	2	7129
Lung	140	63	5	3312

For two microarray data, we use the KMeans-GCSI-MBPSO-ELM approach [30] to perform gene selection, and four and ten genes are selected for the Brain cancer and Lung data, respectively. The number of the hidden nodes in all base ELM is the same, which is 25, 400, 15, 180, 15 and 20 on Diabetes, Satellite Image, Wine, Image Segmentation, Brain cancer and Lung data, respectively. The values for the other parameters are the same as those in the above function approximation problem.

Table 4 shows the mean classification results of the seven algorithms on the six classification problems. The SO-EELM uses the average weighting voting method as the decision rule. From Table 4, the DO-EELM achieves the highest test accuracies among all ensemble of ELM on all data. All ELM ensembles obtain higher test accuracies with less standard deviation than SVM on all data except the Brain cancer data. On the Brain cancer data, SVM achieves higher test accuracy than all ELM ensembles except the DO-EELM. The DO-EELM selects fewer ELM to build the ensemble system than other ensemble methods in all cases. Similarly, the proposed ensemble of ELM gains the better generalization performance with more compact structure than other ELM ensembles and SVM. From [47], the regularized discriminant analysis method also achieved 100% classification accuracy as the DO-EELM on the Wine data. The regularized discriminant analysis method has the potential to increase the power of discriminant analysis in settings for which sample sizes are small and the number of measurement variables is large, while it substantially improves misclassification risk when the population class covariance matrices are not close to being equal and/or the sample size is too small for even linear discriminant analysis to be viable [48]. Since the deficiencies of each ELM may be compensated by the efficiency of the others, the DO-EELM could achieve comparatively high classification accuracy on most data including the Wind data. However, because of its double optimization procedure and being an ensemble method, the DO-EELM requires much more training time than the regularized discriminant analysis method.

**Table 4:** Classification results of the seven algorithms on the six data.

Data	Algorithms	Train accuracy	Test accuracy±Std.	Mean size of ELM ensemble
Diabetes	SVM	0.7807	0.7747±0.0252	/
	E-ELM	0.7886	0.8271±0.0112	12
	EOS-ELM	0.7877	0.8279±0.0109	12
	E-PSOELM	0.8176	0.8316±0.0085	12.35
	E-ARPSOELM	0.8223	0.8359±0.0077	12.7
	SO-EELM	0.8147	0.8367±0.0073	11.05
	DO-EELM	0.8256	0.8536±0.0055	9.6
Satellite Image	SVM	0.8825	0.8689±0.0035	/
	E-ELM	0.9227	0.8927±0.0031	12
	EOS-ELM	0.9232	0.8926±0.0028	12
	E-PSOELM	0.9311	0.8936±0.0031	14.6
	E-ARPSOELM	0.9316	0.8976±0.0023	7.9
	SO-EELM	0.9279	0.9006±0.0023	6.5
	DO-EELM	0.9335	0.9018±0.0022	6
Wine	SVM	0.9973	0.9529±0.0258	/
	E-ELM	0.9875	0.9819±0.0163	12
	EOS-ELM	0.9975	0.9886±0.0087	12
	E-PSOELM	0.9997	0.9888±0.0083	15
	E-ARPSOELM	1	0.9914±0.0088	15.55
	SO-EELM	1	0.9936±0.0087	8.8
	DO-EELM	1	1±0	5.2
Image Segmentation	SVM	0.9393	0.9336±0.0081	/
	E-ELM	0.9722	0.9496±0.0040	12
	EOS-ELM	0.9736	0.9523±0.0035	12
	E-PSOELM	0.9772	0.9530±0.0030	14.85
	E-ARPSOELM	0.9828	0.9562±0.0030	12.7
	SO-EELM	0.9819	0.9575±0.0032	11.6
	DO-EELM	0.9822	0.9665±0.0028	9.3
Brain cancer	SVM	0.8817	0.8368±0.0449	/
	E-ELM	0.9853	0.7336±0.0290	12
	EOS-ELM	0.9676	0.7368±0.0301	12
	E-PSOELM	0.9769	0.7368±0.0273	13.35
	E-ARPSOELM	0.9808	0.7395±0.0118	11.05
	SO-EELM	0.9786	0.7893±0.0207	11.65
	DO-EELM	0.9808	0.8737±0.0106	8.75
Lung	SVM	0.9861	0.9548±0.0165	/
	E-ELM	0.9911	0.9865±0.0093	12
	EOS-ELM	0.9911	0.9873±0.0083	12
	E-PSOELM	0.9896	0.9906±0.0087	15
	E-ARPSOELM	0.9876	0.9960±0.0109	11.35
	SO-EELM	0.9763	0.9921±0.0096	10.8
	DO-EELM	1	1±0	8.3

**Fig 4:**
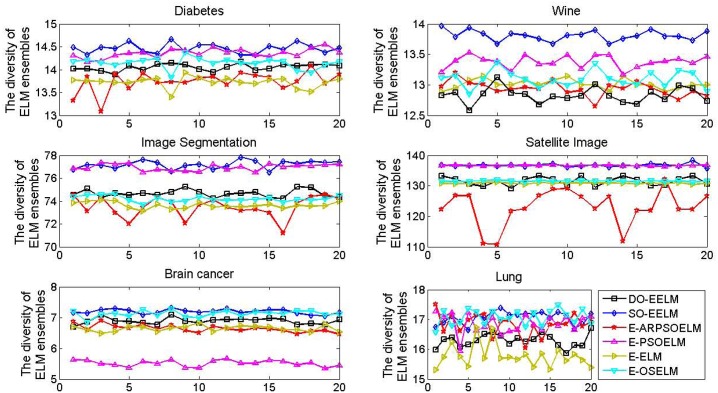
The diversity curves of different ensembles of ELM on the six classification problems with 20 independent runs.

Fig 4 shows the diversity values of different ensemble of ELM on the six classification problems with 20 independent runs. The diversity of the ensemble system built by the DO-EELM is about medium level of those of all ELM ensembles on all data except the Wine data. The diversity value of the ELM ensembles built by DO-EELM is always less than that of the SO-EELM in all cases, and it is the least in most of runs on the Wine data. The DO-EELM has no distinct advantage of absolute diversity over the other ensemble of ELM, because the size of the ensemble system built by the DO-EELM is much less than that of other algorithms.

**Fig 5:**
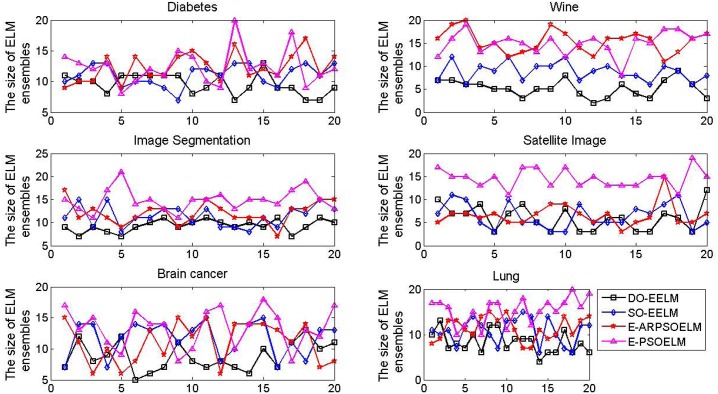
The number of the ELM in the four PSO based ensembles of ELM on the six classification problems with 20 independent runs.

Fig 5 shows the ELM number in the four PSO based ensemble of ELM with 20 independent runs. In most of cases, the proposed method could build an ensemble system with fewer number of ELM than other PSO based ELM ensembles.

### Discussions

**Fig 6:**
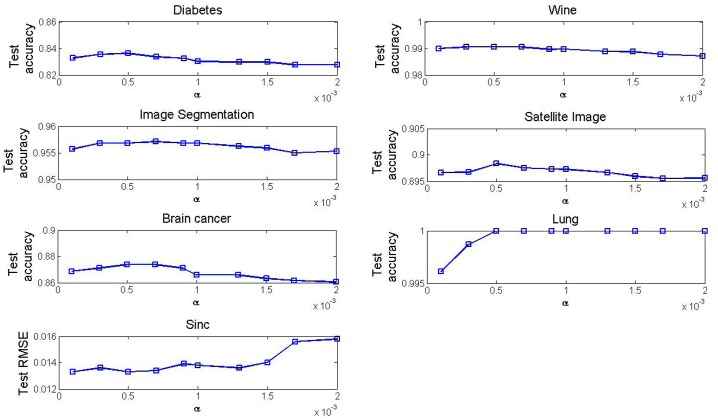
The convergence accuracy vs the different values of the parameter *α* in the DO-EELM method.

Fig 6 depicts the curve of the convergence accuracy as the value of the parameter *α* is selected in the interval of (0, 0.002] in the DO-EELM on the seven data. As for the classification problems, the test accuracy has a slightly downward trend except the Lung data as the value of the parameter *α* increases. As for approximating the Sinc function, the test RMSE has an upward trend as the value of the parameter *α* increases. Form Fig 6, the optimal value of the parameter *α* is 0.0005 in the DO-EELM on all datasets.

**Fig 7:**
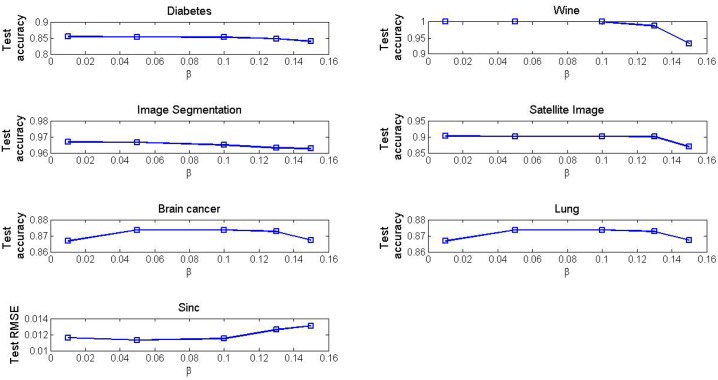
The convergence accuracy vs the different values of the parameter *β* in the DO-EELM method.

Fig 7 depicts the curve of the convergence accuracy as the value of the parameter *β* is selected in the interval of [0.01, 0.15] in the DO-EELM. As the value of the parameter *β* increases, the test accuracy has a downward trend on the four benchmark classification problems. As for two microarray data, the suitable interval of *β* is [0.05, 0.10]. As for approximating the Sinc function, the test RMSE has an upward trend as the value of the parameter *β* increases, especially as the parameter *β* is greater than 0.1.

Figs 6 and 7 provide a guide on how to select the values of the parameters *α* and *β* in the DO-EELM. In general, these parameters should be selected empirically in particular applications.

**Fig 8:**
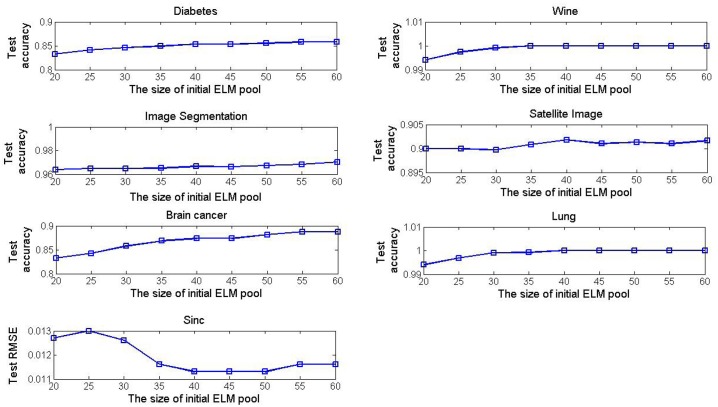
The convergence accuracy vs the size of the initial ELM pool in the DO-EELM method.

Fig 8 shows the effect of the size of the initial ELM pool on the convergence performance in the DO-EELM. In the experiments, all parameters except the size of the initial ELM pool are fixed as their optimal values, the size of the initial ELM pool is select from 20 to 60. The test accuracy has a slightly upward trend on all classification problems as the number of the ELM in the initial ELM pool increases. For approximating the Sinc function, the suitable size of the initial ELM pool is between 40 and 50. From Fig 8, it is a reasonable choice that the size of the initial ELM pool is set as 40 on all data.

**Fig 9:**
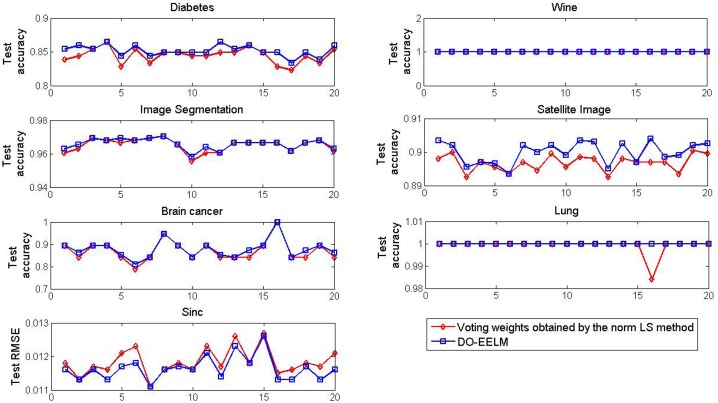
The convergence accuracy of two approaches on the seven data with 20 independent runs.

The optimization of the ensemble weights in the DO-EELM not only reduces the complexity of the ensemble system, but also improves the classification performance of the ensemble system. Fig 9 shows the convergence accuracy between two approaches on the seven data with 20 independent runs. One is the DO-EELM where the initial ensemble weights obtained by the minimum norm LS method are optimized by ARPSO in the second phase, and the other is that the ultimate ensemble weights are obtained by the minimum norm LS method without the further optimization. Two approaches easily achieve 100% test accuracy on the Wine data, while the test accuracy of the DO-EELM is higher than or equal to that of the other approach on the other five classification problems in each run. For function approximation, the DO-EELM achieves the less test RMSE than the other method in most of runs. Therefore, it is necessary to optimize the initial ensemble weights obtained by the minimum norm LS method with ARPSO.

## Conclusions

To establish an effective ensemble of RVFL, the double optimization strategy based on ARPSO was proposed to select the base ELM and determine the ensemble weights in this study. In the first phase, ARPSO selected the optimal base ELM sets by considering the classification performance as well as the diversity of the ensemble of RVFL. In the second phase, the ensemble weights were determined by the minimum norm LS method and ARPSO. Finally, to obtain more compact ensemble system, it was further pruned by deleting the redundant base ELM. Experiment results on the function approximation and six classification problems verified that the proposed approach could gain much higher generalization performance with fewer number of ELM in the ensemble system than some PSO based ensembles of ELM with single optimization and other classical ones. It is evident that the establishment of the initial ELM pool is also an important step in the proposed method. A reasonable initial ELM pool will not only improve the convergence performance of the ensemble system but also decrease the optimization cost. Future work will include how to establish a more effective initial ELM pool and apply the DO-EELM to more complex problems.
